# Establishment of a Novel Platform for Developing Oral Vaccines Based on the Surface Display System of Yeast Spores

**DOI:** 10.3390/ijms26083615

**Published:** 2025-04-11

**Authors:** Chenyu Si, Jiawen Bai, Yuqing Li, Yang Li, Yishi Liu, Xiaoman Zhou, Jie Shi, Hideki Nakanishi, Zijie Li

**Affiliations:** Key Laboratory of Carbohydrate Chemistry and Biotechnology, Ministry of Education, School of Biotechnology, Jiangnan University, Wuxi 214122, China; sichenyu0402@163.com (C.S.); 18538869197@163.com (J.B.); 7210207002@stu.jiangnan.edu.cn (Y.L.); 18662776128@163.com (Y.L.); liuyishi@jiangnan.edu.cn (Y.L.); xiaoman@jiangnan.edu.cn (X.Z.); j.shi@jiangnan.edu.cn (J.S.); hideki@jiangnan.edu.cn (H.N.)

**Keywords:** oral vaccine, spike protein, *S. cerevisiae* spores, surface display, humoral immunity, mucosal immune

## Abstract

Oral vaccines are currently the focus of vaccine development because they are convenient to administer, easy to distribute, and capable of activating mucosal immunity. However, the complexity of the gastrointestinal environment and the lack of delivery vehicles severely limit the stability and effectiveness of oral vaccines. This study established a novel platform for developing oral vaccines based on the surface display system of yeast spores. As a specific example, oral vaccines for COVID-19, designed by displaying the receptor-binding domain (RBD) of the SARS-CoV-2 spike protein on the surface of three *Saccharomyces cerevisiae* spore types, including AN120, *osw2*Δ, and *dit1*Δ, were constructed and evaluated. The displayed RBD showed perfect gastrointestinal stability in vitro and was validated in animal studies to produce effective humoral immunity and significant mucosal immune responses after the vaccination. Notably, the displayed RBD elicited a cellular immune response skewed towards a T-helper type 1 (Th1) cell direction in a mouse model. Our findings proved that the oral vaccines of *S. cerevisiae* spores could rapidly induce a comprehensive and protective immune response to SARS-CoV-2. This study aims to provide a promising and potentially useful system that can be used to develop other oral vaccines.

## 1. Introduction

Vaccines, as key tools for disease prevention and control, have far-reaching implications for healthcare and public health. Compared to injectable vaccines, oral vaccination is convenient, easy to distribute, and capable of activating mucosal immunity, which is currently the focus of vaccine development [1]. However, the complexity of the gastrointestinal (GI) environment and the lack of delivery vehicles severely limit the stability and efficacy of oral vaccines. To address the issue of traditional oral vaccines, oral vaccine technology has continued to advance by using novel platforms such as recombinant bacteria, viruses, and nanoparticle-based delivery systems. Among them, bacteria have the advantages of high immunogenicity, low culture costs, and ease of genome modification, making them ideal chassis strains for oral vaccines [2], which have shown excellent effects in the control of diseases such as cholera and typhoid [3,4]. The spore wall of *S. cerevisiae* contains chitosan, a natural polysaccharide with demonstrated adjuvant properties that can enhance both mucosal and systemic immune responses, also exhibits excellent biocompatibility, biodegradability, and safety profiles while effectively potentiating vaccine efficacy through multiple mechanisms [5]. This intrinsic adjuvant activity, particularly valuable in oral vaccine design as shown in aquatic vaccine studies [6,7,8], may synergize with our platform’s antigen-display capability to promote robust humoral and cellular immunity.

The target proteins or antigens can be expressed and anchored to the surface of microbial cells, which have relatively independent spatial structures and biological activities [9], using the recombinant technique. The Baker’s yeast of *Saccharomyces cerevisiae* has Generally Recognized as Safe (GRAS) status and is widely utilized in pharmaceutical, agricultural, and food industries [10]. Under starvation conditions, *S. cerevisiae* diploid cells stop vegetative growth and start the sporulation process. The structure of the yeast spore wall contains four layers: mannan, β-glucan, chitosan, and dityrosine layers (from the inner layer to the outer layer in a sequential manner). It is worth noting that the dityrosine and chitosan layers are special components of the spore wall, which is different from the vegetative yeast cell wall. The dityrosine layer can serve as a barrier to restrain the diffusion of soluble proteins and resist diverse environmental pressures, such as degradation enzymes, high temperatures, and organic reagents [11,12]. There are three types of *S. cerevisiae* spores according to the structural differences of the spore wall. We systematically evaluated mutants with defined outer wall deficiencies: wild-type AN120 spores possess intact dityrosine layers; *osw2*Δ spores show structurally loose dityrosine layers due to *OSW2* gene knockout; and *dit1*Δ spores completely lack the outermost dityrosine layer (with exposed chitosan) due to disruption of the *DIT1* gene essential for dityrosine synthesis [13].

In this study, a type of oral vaccine was generated in which the receptor-binding domain (RBD) of the spike protein which is selected as the important target of SARS-CoV-2 was displayed on the surface of *S. cerevisiae* spores [14,15,16,17]. This novel oral vaccine was stable in the gastrointestinal tract, and the immunological effects and reactogenicity were assessed. Furthermore, the oral vaccination could induce broadly neutralizing antibodies and durable protective immunity against the infection of SARS-CoV-2.

## 2. Results

### 2.1. Expression and Localization of RBD on the Surface of S. cerevisiae Spores

**Figure 1:**
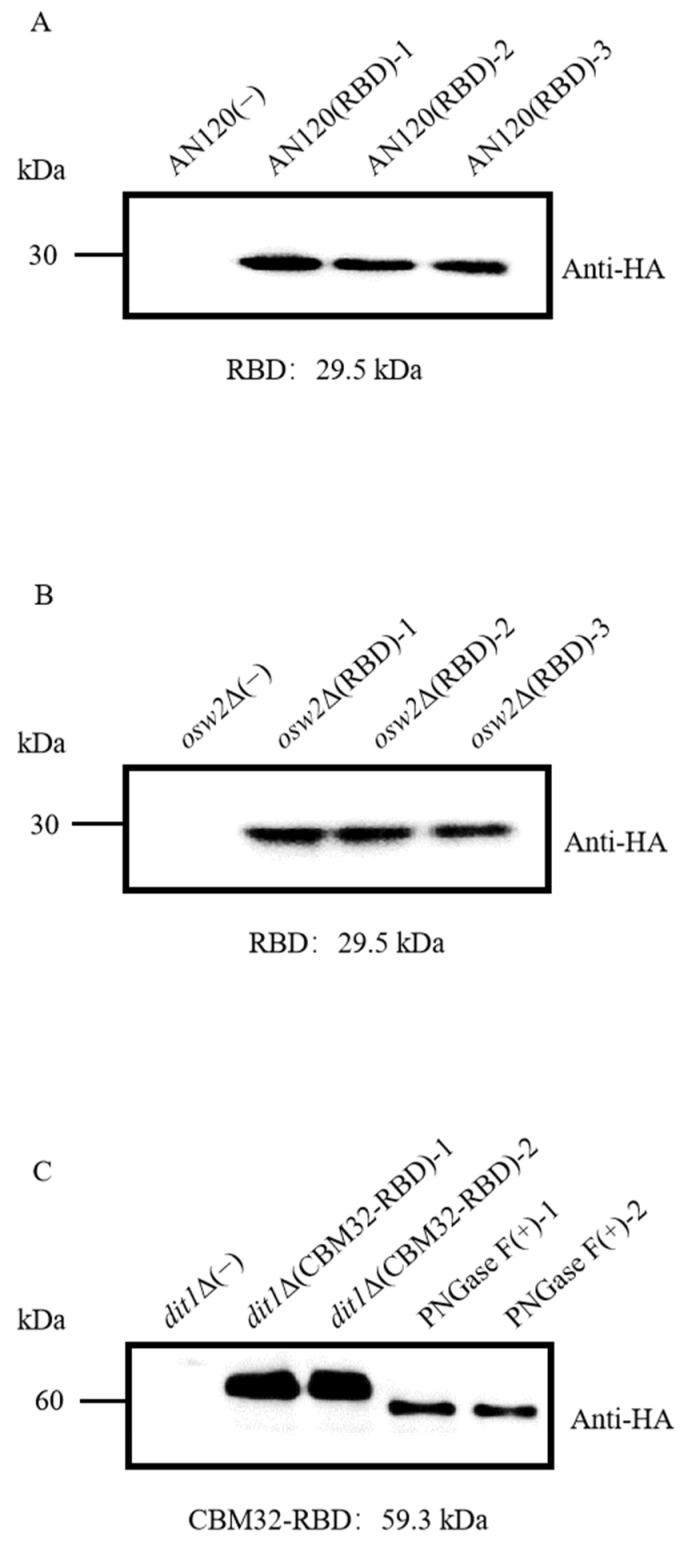
Western blot analysis of indicated spores expressing RBD-HA or CBM32-RBD-HA using anti-HA antibodies. (**A**) Western blot analysis of RBD expression in AN120 spores. (**B**) Western blot analysis of RBD expression in *osw2*Δ spores. (**C**) Western blot analysis of CBM32-RBD expression in *dit1*Δ spores treated with the PNGase F enzyme.

To express the RBD of the S protein on the surface of wild type (AN120), *osw2*Δ, and *dit1*Δ spores, the secreted form of RBD labeled with the hemagglutinin (HA; YPYDVPDYA) epitope tag was constructed, and the expression level of RBD was detected by western blot. The result showed that RBD with the expected molecular weight (29.5 kDa) could be detected in the AN120/pRS306-*ss-RBD-HA* and *osw2*Δ/pRS306-*ss-RBD-HA* spores (Figure 1A,B). However, for *dit1*Δ/pRS306-*ss-CBM32-RBD-HA* spores, the protein molecular weight was higher than the theoretical value (59.3 kDa), indicating that CBM32-RBD-HA was possibly glycosylated. It was predicted that the amino acid sequence of CBM32 contained five N-glycosylation sites, and the glycosylation of CBM32 could be confirmed by removing the N-glycan hydrolyzed by PNGase F. As expected, the molecular weight of CBM32-RBD was reduced to the theoretical value after removing the N-glycan (Figure 1C). In contrast, no specific band was observed for AN120, *osw2*Δ, and *dit1*Δ spores without RBD expression.

**Figure 2:**
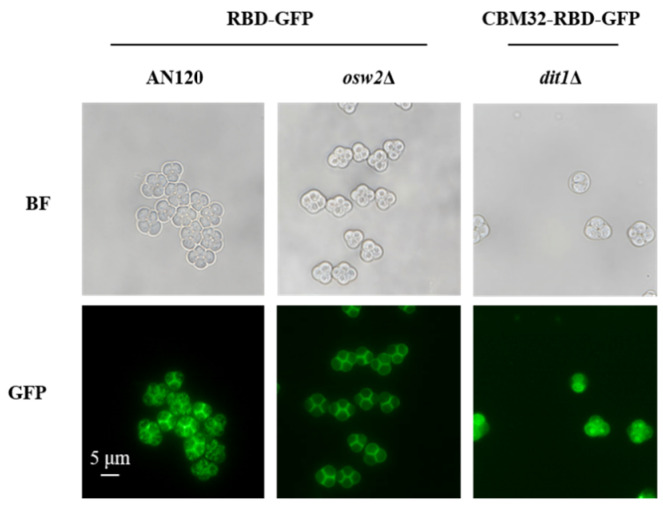
Localization determination of RBD on the surface of indicated spores (AN120, *osw2*Δ, and *dit1*Δ) expressing RBD-GFP or CBM32-RBD-GFP under fluorescent (GFP) or bright-field (BF) microscopy. Scale bar, 5 µm. All the images of fluorescence microscopy were acquired under the same imaging conditions.

To determine the localization of RBD by fluorescence microscopy, the fusion expression of RBD with green fluorescent protein (GFP) was conducted in AN120/pRS306-*ss-RBD-GFP*, *osw2*Δ/pRS306-*ss-RBD-GFP*, and *dit1*Δ/pRS306-*ss-CBM32-RBD-GFP* spores. As illustrated in Figure 2, green fluorescence could be observed around the surface of the spore wall. It should be noted that the fluorescence of *dit1*Δ spores lacking the outermost dityrosine layer was not obviously diffused into the cytoplasm of the spore ascus due to the presence of CBM32 anchor protein. Taken together, the above results proved that the RBD was successfully displayed on the surface of the spore wall, which formed the basis for the subsequent investigation of the vaccine immunization effect of this oral vaccine display system.

### 2.2. Stability of Spores by Stimulated Gastrointestinal Digestion

**Figure 3:**
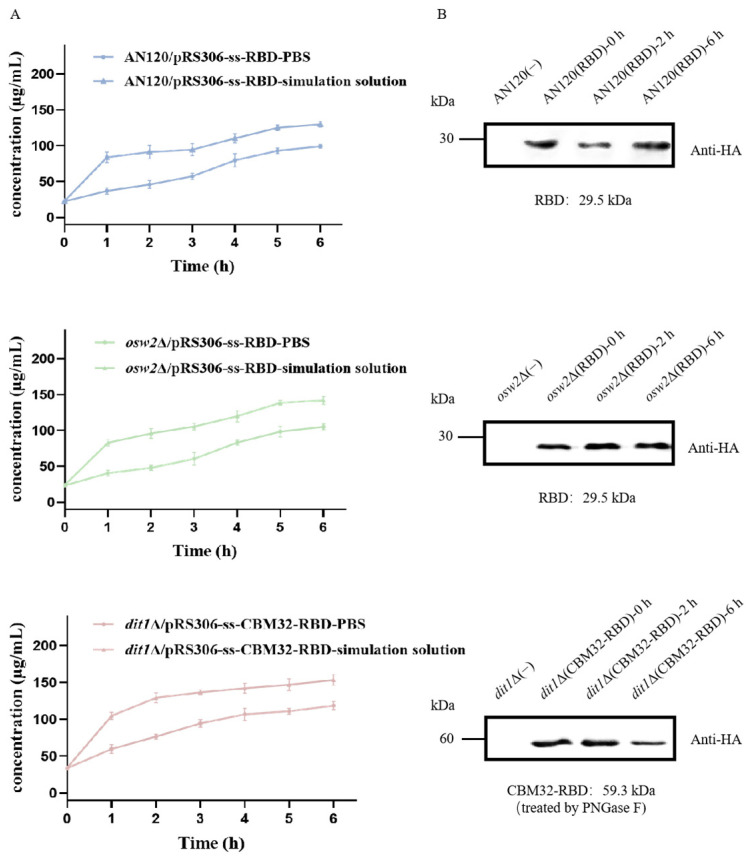
Evaluation of protein stability during in vitro gastrointestinal digestion. (**A**) Total protein released from the spores digested after 1, 2, 3, 4, 5, and 6 h. (**B**) Amount of RBD from the spores after 0, 2, and 6 h treatment with gastrointestinal simulant.

The BCA assay results demonstrated that the total protein released over 6 h was low with no significant trend (Figure 3A). Furthermore, the expression level of RBD in the spores after 0, 2, and 6 h of treatment with the gastrointestinal simulation fluid was determined by western blot. It was found that there was no obvious change in the amount of RBD (Figure 3B), which proved that the oral vaccine constructed by the spore surface display system had perfect stability and protected the antigen from gastrointestinal invasion.

### 2.3. Measurement of the SARS-CoV-2 Antibodies

**Figure 4:**
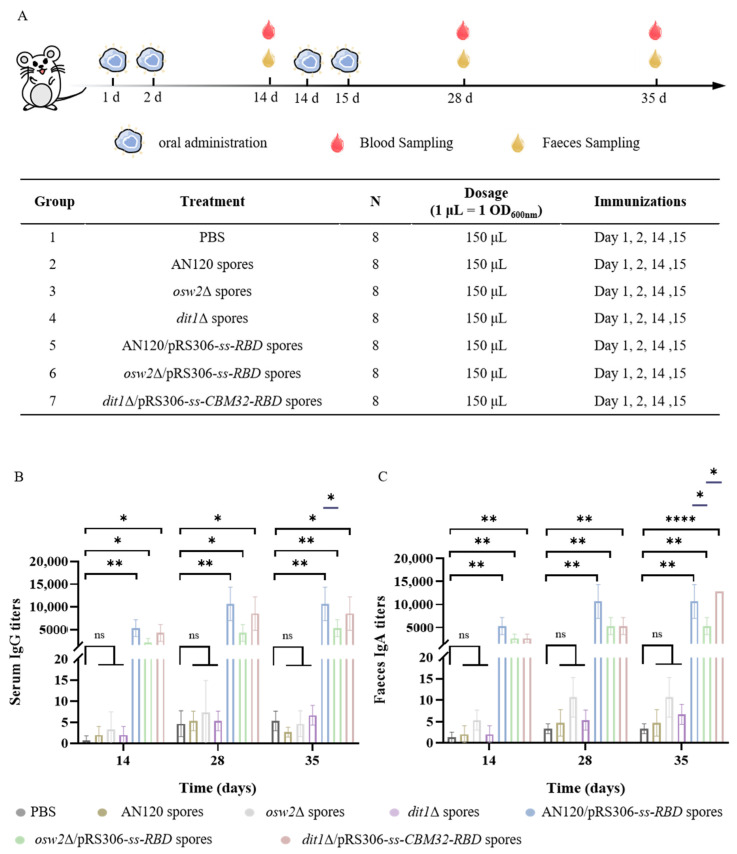
Protective immune responses. (**A**) Schematic of the immunization regimen. (**B**) anti-RBD IgG titers on day 14, 28, and 35 following initial immunization. (**C**) anti-RBD IgA titers on day 14, 28, and 35 following initial immunization. Data were the geometric means ± geometric SD. * *p* < 0.05; ** *p* < 0.01; **** *p* < 0.0001; ns, not significant.

After oral primary immunization of the mice, serum and feces were collected on day 14, 28, and 35 to assess the humoral and mucosal immune responses (Figure 4A). It was found that the levels of IgG antibodies elicited by oral vaccines were superior to the control groups (Figure 4B). The IgG titers elicited by oral vaccines at post-boost immunization (day 28) were nearly 2-fold than the primary immunization (day 14) in AN120/pRS306-*ss*-*RBD*, *osw2*Δ/pRS306-*ss-RBD* and *dit1*Δ/pRS306-*ss*-*CBM32-RBD* groups, respectively. Moreover, the titers of IgG remained stable and had no significant change on day 35 compared with day 28. It was worth noting that groups AN120/pRS306-*ss-RBD* and *dit1*Δ/pRS306*-ss-CBM32-RBD* demonstrated higher amounts of IgG antibodies than group *osw2*Δ/pRS306-*ss-RBD* on day 35. In particular, the titer of AN120/pRS306-*ss-RBD* was remarkably higher than that of group osw2Δ/pRS306-*ss-RBD*, which represented better immune persistence. Moreover, the negligible anti-RBD IgG titers in empty spore (AN120, *osw2*Δ, and *dit1*Δ) control groups were observed and these values were >500-fold lower than vaccinated groups. A similar trend was also observed for IgA antibodies, and higher IgA titers against RBD were detected in AN120/pRS306-*ss-RBD* and *dit1*Δ/pRS306*-ss-CBM32-RBD* groups (Figure 4C). These results indicated that the oral vaccine for SARS-CoV-2 virus could effectively produce antibodies during the immune cycle through the *S. cerevisiae* spore display system and maintain antibody levels for up to 5 weeks, which was consistent with the key characteristics of the vaccine to protect the body during the initial phase of exposure to the virus or antigen.

### 2.4. Th1-Skewing of the T Cell Response

**Figure 5:**
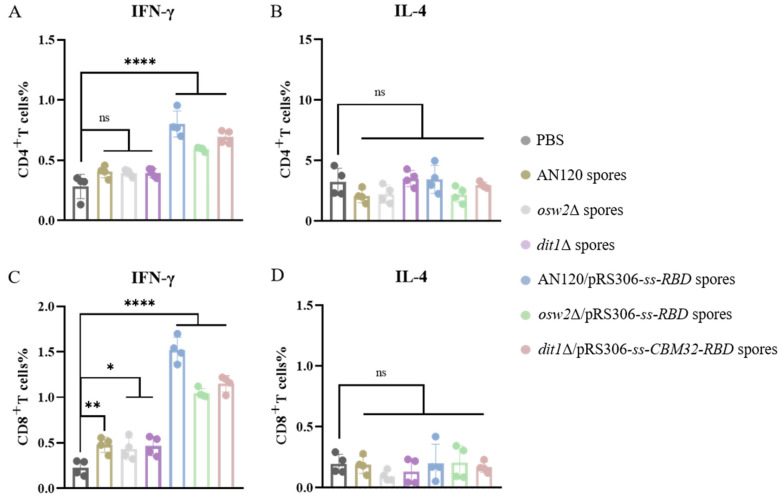
Intracellular cytokine analysis. (**A**) Percentage of CD4^+^ IFN-γ cells. (**B**) Percentage of CD4^+^ IL-4 cells. (**C**) Percentage of CD8^+^ IFN-γ cells. (**D**) Percentage of CD8^+^ IL-4 cells. Data were the geometric means ± geometric SD. * *p* < 0.05; ** *p* < 0.01; **** *p* < 0.0001; ns, not significant.

The cellular immune response is the key to the function of vaccines, which can promote phagocytosis and the elimination of pathogens against foreign invaders. To assess the specific T cell response induced by the constructed oral vaccine based on the spore surface display system, the spleens of mice were removed on day 35 after the above-mentioned administration. The single-cell suspension of splenocytes was prepared for culture in vitro and stimulated with the recombinant RBD. Flow cytometry staining of intracellular factors (IFN-γ and IL-4) showed a strong T cell response to SARS-CoV-2 in all immune groups (Figure 5). Further analysis of the Th bias of the oral vaccine-induced T cell response was performed. The increase of T-helper type 1 (Th1) cytokine (IFN-γ) levels in the oral vaccine groups compared with the control group (PBS) and the empty spore groups were observed (Figure 5A,C). In contrast, the levels of Th2 cytokine (IL-4) hardly fluctuated (Figure 5B,D). These results demonstrate that the COVID-19 oral vaccine developed using the *S. cerevisiae* spore surface display system preferentially elicits Th1-biased immune responses in splenic lymphocytes, indicating robust systemic cell-mediated immunity. While mucosal-associated lymphoid tissues (MALT) may exhibit distinct immune polarization patterns, the splenic data unequivocally establish the vaccine’s capacity to stimulate systemic cellular immunity. Crucially, the observed mucosal IgA production (Figure 4C) demonstrates complementary local immune protection. Together, these findings confirm that the spore-based delivery system induces comprehensive immune activation across both systemic and mucosal compartments.

### 2.5. Antibody Neutralizing Activity

**Figure 6:**
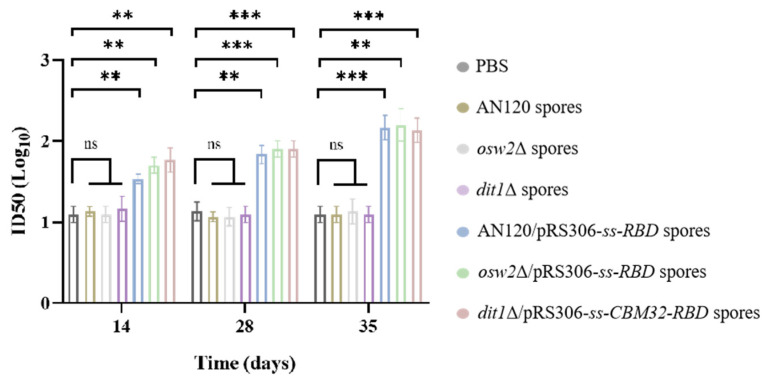
SARS-CoV-2 pseudovirus neutralization evaluation. Neutralizing antibody titers were detected after oral administration. Data were the geometric means ± geometric SD. ** *p* < 0.01; *** *p* < 0.001; ns, not significant.

The antiserum was assessed for the ability to neutralize SARS-CoV-2 pseudovirus. In Figure 6, none of the serum samples from the empty spore groups (AN120, *osw2*Δ, and *dit1*Δ) had neutralization activity, which was basically the same as that in the PBS control group. On day 14 and 28 after the first immunization, the titers of oral vaccine were below 1:80. However, the titers continued to rise after the second immunization until they reached approximately 1:158, 1:158 and 1:126, respectively, in AN120/pRS306-*ss-RBD*, *osw2*Δ/pRS306-*ss-RBD*, and *dit1*Δ/pRS306*-ss-CBM32-RBD* groups on day 35 after the first immunization. Collectively, these results demonstrated that the COVID-19 oral vaccine could induce neutralization antibodies against SARS-CoV-2 pseudovirus infection.

### 2.6. Reactogenicity Studies In Vivo

**Figure 7:**
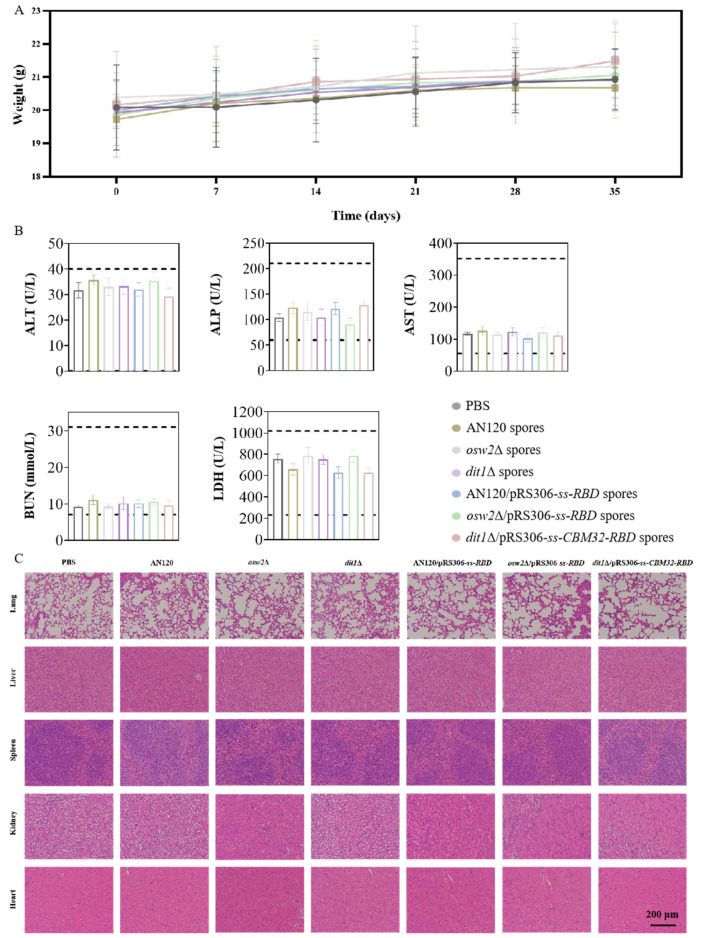
Reactogenicity evaluation of mice. (**A**) Body weight gain in mice. (**B**) Biochemical analysis of the blood of BALB/c mice. Indicators of hepatic function: ALT (alanine aminotransferase); ALP (alkaline phosphatase); and AST (aspartate aminotransferase), renal function: BUN (urea nitrogen), and cardiac function: LDH (lactate dehydrogenase). (**C**) Biocompatibility evaluations via H&E staining of vital organ sections from BALB/c mice. Scale bar represents 200 µm.

To further evaluate the reactogenicity of this type of oral vaccine, the reactogenicity assessment was conducted. During the experimental process, none of the animals died and their body weights were steadily increased (Figure 7A). The liver function was reflected by the activities of alanine aminotransferase (ALT), alkaline phosphatase (ALP), and aspartate aminotransferase (AST), and the function of heart and kidney was assessed by the level of urea nitrogen (BUN) and lactate dehydrogenase (LDH). According to the blood biochemical index values measured, there were no differences between the oral vaccine groups and the PBS group (Figure 7B). Furthermore, no significant pathological changes were observed in vital organs such as the lungs, livers, spleens, kidneys and hearts on day 35 (Figure 7C). These results demonstrated that the COVID-19 oral vaccine showed good reactogenicity and has the potential for clinical application.

## 3. Discussion

The use of oral vaccines is hampered by the antigens degradation in the acidic environment of the gastrointestinal tract (GI). Hence, it is very important to provide a safe and innovative vehicle for the efficient delivery of oral protein vaccines by overcoming the harsh GI environment. Yeast has been widely used as an oral vaccine carrier due to its rapid growth, low cost, and non-pathogenicity. Various viral antigens were expressed in yeast as potential vaccines [18], and impressive progress has been achieved in vaccine applications against hepatitis B virus, influenza virus [19], and encephalitis virus [20].

Currently, the technique of expressing RBD using yeast or displaying RBD on the yeast surface has been validated in the fight against COVID-19 infection [21]. However, displaying viral antigens on the surface of yeast spores for the exploration of oral vaccines has never been reported. Compared with vegetative yeast cells, *S. cerevisiae* spores have a smaller size and unique cell wall, exhibiting better tolerance to diverse environmental stresses including enzyme digestion and acidic conditions. In this study, a novel oral vaccine based on the display of RBD of spike protein on the surface of *S. cerevisiae* spores was developed. RBD was efficiently displayed on the surface of three types of spores (AN120, *osw2*Δ or *dit1*Δ) and had perfect gastrointestinal stability. Importantly, this oral vaccine could evoke effective humoral immunity and significant mucosal immune responses.

The collaborative action of virus-specific humoral and cellular immune responses is helpful for the host fighting against viral infection. In intestinal immunity, the development of IgA^+^ and IgG^+^ B cells are induced by activated T cells in the germinal center of the mucosal-associated lymphoid tissue (MALT) [22]. Specific IgG for SARS-CoV-2 could be measured 7–14 days after the onset of symptoms [23], and for most vaccines, the antibody level can reach the peak after the secondary immunity [24]. After the second immunization, IgG titers (Figure 4B) and IgA titers (Figure 4C) increased almost 2-fold and stabilized over the following days with good immune persistence. By comparison with the empty spore groups, better humoral and mucosal immunity was demonstrated after the administration of the oral vaccines of spores. It is worth noting that antigen-specific IgA is essential for protecting gastrointestinal, respiratory, and urogenital surfaces, and preventing opportunistic infections. It would appear that a small amount of immune effect is generated in empty spores; we wonder if this may be due to the fact that the spores themselves have some immune effect. However, this study did not assess whether the mucosal immunity elicited by the oral vaccine provides better immune protection against SARS-CoV-2 compared to current mRNA vaccines, and this comparison should also be added in future experiments.

Increasing evidence indicates that cellular immune response plays a crucial part in clearing SARS-CoV-2 and mitigating COVID-19. Cytokines are generated by activated immune or non-immune cells including CD4^+^ T and CD8^+^ T cells, which can regulate immune and inflammatory responses. The IFN-γ is produced by Th1 cells and is involved in cellular immune responses, while IL-4 is generated by Th2 cells and induces humoral immune responses. Following oral administration of the vaccine, the level of IFN-γ was increased significantly, and no significant rising trend for IL-4 level was observed. These results suggested that this type of oral vaccine tended to induce a Th1-type cellular immune response. While splenic T cell analysis revealed a Th1-dominant response, the concurrent production of IgA—typically associated with Th2 responses—suggests compartmentalized immune activation. This likely reflects differential responses in systemic (spleen, Th1) versus mucosal (gut-associated lymphoid tissue, IgA) compartments. Importantly, this dual response profile indicates our oral spore vaccine can simultaneously engage both humoral (IgA) and cellular (Th1) arms of immunity, potentially offering more comprehensive protection.

Virus neutralization assay is critical to evaluate the effects of vaccines and immunotherapy, as well as investigating the immune response, diffusion, and pathogenic mechanism of the disease. The infectious virus can be replaced by the pseudovirus to perform the virus neutralization activity. SARS-CoV-2 pseudovirus contains only the spike structural protein, and the infection leads to a single round of replication, thus allowing for the experiment to be conducted safely [25]. The neutralization assay of pseudovirus showed that the serum samples of orally immunized mice had strong neutralization activities. Moreover, the titers increased over time, demonstrating that the oral vaccine also has good immune persistence. A follow-up challenge study using authorized BSL-2 animal models (e.g., hACE2 transgenic mice) is planned.

Taken together, all the experimental results proved that the oral vaccine developed based on the surface display platform of yeast spores can exert good immunotherapeutic effects. Importantly, there was no adverse reaction after vaccination in mice, suggesting that the oral vaccine prepared by this system had good safety. It was speculated that the better immunity of an oral vaccine produced by AN120 spores may be due to the fact that the outermost dityrosine layer is effective in resisting the stresses of the external environment and protecting the antigen. For the oral vaccine produced by *dit1*Δ spores, the exposed chitosan layer may function as a natural adjuvant, similar to other immunostimulatory polysaccharides. Chitin and its derivatives are known to enhance immune responses through pattern recognition receptors like TLR-2 and Dectin-1 [26,27]. Our study focused on the peak and early maintenance of antibody responses (up to day 35). While this timeframe aligns with standard vaccine efficacy studies [28,29], longer-term evaluations will be essential to fully characterize the durability of this platform. Future studies will include timepoints beyond 8 weeks to assess sustained immunogenicity. A key limitation is the lack of comparison to licensed COVID-19 vaccines, precluding efficacy benchmarking. However, our mucosal immunization strategy (evidenced by fecal IgA in Figure 4C) represents a distinct advantage over injectable platforms. Meanwhile, previous studies showed that bivalent COVID-19 vaccines induced some cross-reactive immune responses against SARS-CoV-2 variants [30,31], and multi-antigen vaccines have many clinical applications, such as human papillomavirus, HIV, and other infectious diseases caused by influenza [32,33]. In the future, a multi-antigen vaccine based on the *S. cerevisiae* spore surface display platform will be developed and the efficacy of this type of oral vaccine will be assessed.

## 4. Materials and Methods

### 4.1. Construction of Recombinant Strains and Plasmids

The *Escherichia coli* TOP10 strain was used for plasmids amplification and construction. All the *S. cerevisiae* strains in a fast-sporulating *SK-*1 genetic background were adopted for the sporulation in this study. RBD-L452K-F490W (hereafter referred to as RBD) sequence was selected, which could raise an enhanced immune response in mice relative to the Wuhan-Hu-1 sequence [34,35]. To achieve the display of RBD on the *S. cerevisiae* spore surface, RBD was fused with the signal peptide sequence (ss) derived from the Spr1 protein (Amino acid sequence: MVSFRGLTTLTLLFTKLVNCN) at the N-terminus and ligated into the pRS306 plasmid containing a TEF1 promoter. Strains and plasmids used in this study were shown in Figures S1 and S2. The RBD fragment was first amplified by PCR with primers and the product was inserted into the plasmid pRS306 to generate the recombinant plasmid pRS306-*ss-RBD*. Next, the plasmid pRS306-*ss-RBD* was linearized and transformed into competent *S. cerevisiae* AN120 and *osw2*Δ haploid cells (a and α type), respectively. The positive colonies of AN120/pRS306-*ss-RBD* and *osw2*Δ/pRS306-*ss-RBD* haploid cells were screened by SD-U Amino Acid-Deficient Medium (0.67% YNB, 2% agar powder, 2% glucose, 0.2% Amino acid mixture powder without Ura), and diploids were next screened by SD-LA Amino Acid-Deficient Medium (0.67% YNB, 2% agar powder, 2% glucose, 0.2% Amino acid mixture powder without Leu and Arg).

To determine the orientation of RBD by fluorescence microscope, the green fluorescent protein (GFP) gene was inserted into the C-terminus of the RBD to obtain the recombinant plasmid pRS306-*ss-RBD-GFP*. To assay the amount of RBD by western blot, hemagglutinin (HA) was tagged to the C-terminus of RBD to obtain the recombinant plasmid pRS306-*ss-RBD-HA*. The diploid *S. cerevisiae* recombinant strains AN120/pRS306-*ss-RBD-GFP*, *osw2*Δ/pRS306-*ss-RBD-GFP*, AN120/pRS306-*ss-RBD-HA*, and *osw2*Δ/pRS306-*ss-RBD-HA* were obtained using a similar method as above. To enhance the display efficiency of RBD in *S. cerevisiae dit1*Δ spores, the sequence of the carbohydrate-binding module CBM32 derived from *Paenibacillus sp. IK-5*, *Pb chitosanase* (GenBank: BAB64835.1) was inserted into the N-terminus of the RBD protein to obtain the recombinant strains *dit1*Δ/pRS306-*ss-CBM32-RBD*, *dit1*Δ/pRS306-*ss-CBM32-RBD-GFP*, and *dit1*Δ/pRS306-*ss-CBM32-RBD-HA* [36]. Strains, plasmids, and primers related in this research are summarized in Tables S1–S3, respectively.

### 4.2. Production and Purification of Spores

Yeast spores were prepared as follows: First, yeast cells from a single colony were cultivated overnight in 5 mL of YPAD medium (0.5% yeast extract, 1% peptone, 2% glucose, 0.003% Adenosine) at 30 °C. For the induction of sporulation, 5 mL of the culture broth was then transferred to 100 mL of YPACE medium (1% yeast extract, 2% peptone, 2% potassium acetate, 0.003% Adenosine) and cultivated for 24 h. The cells were collected by centrifugation and cultured in 50 mL of 2% potassium acetate for 48 h. The efficiency of sporulation was examined under the light microscopy. To obtain the single spores, the ascospores were resuspended in 5 mL of spheroplast buffer (1.4 M sorbitol, 40 mM-mercaptoethanol, 50 mM potassium phosphate, pH 7.5) and digested by lyticase from *Arthrobacter luteus* (Sigma-Aldrich, St. Louis, MO, USA). After incubation at 37 °C for 3 h, the ascospores were rinsed twice using the spheroplast buffer. Then, the ascospores were resuspended in 5 mL of fresh spheroplast buffer and sonicated to disrupt the ascus membrane for 10 min. The single free spores were examined by the microscope. Finally, the spores were washed three times using precooled sterilized water, and the purified spores were freeze-dried and stored at −20 °C.

### 4.3. Microscopy and Western Blot Analysis

The fluorescence image for the localization of RBD was acquired using a Nikon Eclipse Ti-E inverted microscope (Nikon, Tokyo, Japan) furnished with a DS-Ri1 camera (Nikon, Tokyo, Japan) and NIS-Element AR software 4.3. To carry out the western blot assay, the isolated spores were first suspended in 200 µL of protein extraction buffer (0.2 M D-sorbitol, 50 mM Tris-HCl, 1 mM EDTA, pH 7.5) and 2 µL of protease inhibitor cocktail (MedChemExpress, Monmouth Junction, NJ, USA), then treated with a freeze-grinding instrument for 30 min. To determine the glycosylation level of RBD, the supernatant was treated with PNGase F (Adamas Life, Shanghai, China) to release the glycan [37], and the supernatant was collected and detected by SDS-PAGE. The gel was then transferred to a polyvinylidene difluoride (PVDF) film (0.45 µm). After blocking with 5% non-fat milk at room temperature for 2 h, the film was first incubated with 1:5000 diluted mouse anti-HA antibody (TransGen Biotech, Beijing, China) at 4 °C for 2 h. Then, the film was incubated with the secondary antibody horseradish peroxidase (HRP)-conjugated goat anti-mouse IgG (TransGen Biotech, China) with a dilution of 1:5000 at room temperature for 1 h. The Clarity Western ECL substrate (Bio-Rad, Hercules, CA, USA) was used to visualize the signals, and the images were acquired by ImageQuant LAS 4000 (GE Healthcare Bio-Science, Danderyd, Sweden).

### 4.4. Stimulated Gastrointestinal Digestion of Spores Displaying RBD In Vitro

To evaluate the gastrointestinal stability of AN120/pRS306-*ss-RBD*, *osw2*Δ/pRS306-*ss-RBD,* and *dit1*Δ/pRS306-*ss-CBM32-RBD* spores displaying RBD, the spores were incubated in simulated gastric fluid (SGF, pH 2.0) for 2 h and simulated intestinal fluid (SIF, pH 7.4) for 4 h at 37 °C, 100 rpm, respectively. First, 20 mg of spores were dispersed in 20 mL of SGF, stirred at 37 °C for 2 h, then spores were collected by centrifugation at 8000 rpm for 10 min, and transferred to 20 mL of SIF and kept stirring for 4 h. The supernatant was sampled at the time points: 1, 2, 3, 4, 5, and 6 h, and supplemented with the same volume of fresh buffer. The amounts of proteins released from spores were measured by BCA assay. The technique used for RBD detection in gastrointestinal assays was the western blot assay. Equal amounts of 20 mg of spores were taken and treated in 20 mL of simulation solution for 2 h and 6 h. Treated spores were centrifuged at 10,000 rpm for 10 min, the precipitate was harvested and then subjected to western blot assay using anti-HA tag antibody.

### 4.5. Vaccine Formulation and Oral Immunization

AN120, *osw2*Δ, *dit1*Δ, AN120/pRS306-*ss-RBD*, *osw2*Δ/pRS306-*ss-RBD*, and *dit1*Δ/pRS306-*ss-CBM32-RBD* spores were thermally inactivated at 60 °C for 1 h, then the final amounts of spores were adjusted to 1.0 OD_600_/µL. Female specific pathogen-free (SPF) BALB/c mice (8-week-old, 14–17 g) purchased from SPF Biotechnology were maintained by the SPF animal experiment center of Jiangnan University. After an adaptive feeding period of 7 days, mice were randomly divided into 7 groups (*n* = 8 for each group) and immunized with different vaccination regimens. The mice were orally administered 150 µL (1 µL = 1 OD_600nm_) of six kinds of spores for initial immunization on day 1 and 2, and for enhanced immunization on day 14 and 15. Equal dose of PBS was adopted as a control. The samples of blood and feces were harvested from vaccinated mice on day 14, 28 and 35 after the initial immunization, sacrificed after 35 days. The whole blood of mice was collected and coagulated at room temperature for 2 h. Serum was isolated by centrifugation at 3000 rpm for 10 min and deposited at −20 °C for use. Fecal pellets (30 mg/mouse) were mixed with 300 µL of sterile PBS. The supernatants of suspension liquid were harvested by centrifugation at 2000 rpm for 10 min and deposited at −20 °C before processing.

### 4.6. Enzyme-Linked Immunosorbent Assay (ELISA)

The RBD-specific antibodies associated with antigen-specific IgG levels in mouse serum and IgA levels in mouse feces were determined using ELISA. In brief, 100 µL of diluted RBD protein (2 µg/mL) was coated in flat-bottom 96-well plates and incubated at 4 °C overnight. After incubation, coated wells were rinsed thrice with Tris-NaCl buffer in the presence of 0.05% Tween 20 (TBST) and then blocked with 1% bovine serum albumin (BSA) in TBST at 37 °C for 1 h. After washing four times with TBST, diluted samples (starting dilution 1:100, serial dilutions of 2-fold) from immunized animals were added to the wells at 37 °C for 1 h. Subsequently, HRP-conjugated goat anti-mouse IgG (1:5000) (TransGen Biotech, China) and HRP-conjugated goat anti-mouse IgA (1:10,000) (ImmunoWay, San Jose, CA, USA) were added to the plates, respectively, which were then incubated at 37 °C for 1 h. Thereafter, the plates were rinsed with TBST, and the value of optical density was detected at 450 nm by a microplate reader using EL-TMB Chromogenic Reagent kit (Sangon Biotech, Shanghai, China).

### 4.7. Preparation of Splenocytes

The spleens of mice were harvested on day 35 after the initial vaccination. Single-cell suspensions were prepared by dissociation through a 70 µm screen, and cells were obtained by centrifugation at 1500 rpm for 5 min. Red blood cells (RBCs) were removed by incubation with the lysis buffer of ammonium-chloride-potassium (ACK) (Solarbio, Beijing, China) at room temperature for 5 min. Then, the splenocytes were suspended in PBS for enumeration. After the centrifugation, the pelleted cells were resuspended in Roswell Park Memorial Institute (RPMI) 1640 medium containing 10% *v*/*v* fetal bovine serum (VivaCell, Shanghai, China) and penicillin streptomycin (Beyotime Biotech, Beijing, China).

### 4.8. Flow Cytometry Analysis

Initially, 1 × 10^6^ isolated spleen cells were transferred to 96-well plates and 2 µg/mL of recombinant RBD (10 µg/mL) was added for the stimulation at a humidified 5% CO_2_ incubator for 36 h. Afterwards, cells were washed with stain buffer (Proteintech, Rosemont, IL, USA) thrice, and stained with CD4-ABflo 488, CD8a-ABflo 647, (ABclonal Technology, Wuhan, China) at 4 °C for 1 h. Intracellular staining was finished after membrane staining of cells. The cells were washed twice, then Foxp3/Transcription Factor Staining Buffer Set (Thermo Fisher Scientific, Waltham, MA, USA) was used for the fixation and permeabilization [38], and labeled with IFN-γ-PE-Cy7, IL-4-PE (BD Biosciences, Franklin Lakes, NJ, USA) for 1 h at 4 °C [39]. Next, cell pellets were washed and resuspended in stain buffer prior to FACS acquisition and analysis using Flowjo software 10.8.1 (BD FACSArialll, Piscataway, NJ, USA).

### 4.9. Pseudovirus Neutralization Antibody Assay

The target cells (HEK293T-ACE2) [40] were incubated in a 96-well plate overnight, 1 × 10^4^ cells per well. Serum samples were inactivated at 56 °C for 30 min, then mouse serum pooled from individual mice within each group were serially diluted 2-fold and incubated with a predetermined amount of SARS-CoV-2 pseudovirus (Yeasen Biotechnology, Shanghai, China). After 1 h, the mixed liquid was added into a 96-well plate of HEK293T cells stably expressing hACE for a night. After 72 h infection, the amount of pseudovirus entering the target cells was calculated to obtain the neutralizing antibody concentration of the sample [41,42] by measuring the expression level of luciferase. The relative luminescence units (RLU) were detected based on the Dual-Luciferase Reporter Assay System (Promega, Madison, WI, USA). The cell control (CC) with only cells and the virus control (VC) with viruses and cells were set up in each plate. The half maximal effective concentration (EC50) was determined for the samples.

### 4.10. Reactogenicity Assessment

Biochemical analyzers were adopted to measure systemic toxicity in mice by determining the serum indicators of cardiac, hepatic, and renal functions. The serum indexes of heart, liver, and kidney function and histological changes of vital organs were measured by biochemical analyzer. Further, the lungs, livers, spleens, kidneys, and hearts were isolated and stained with hematoxylin-eosin (H&E). The weights of the mice were monitored and recorded once per week during rearing.

### 4.11. Statistical Analysis

The statistical analysis was performed using GraphPad Prism 9.0 software version. All data were shown as mean ± standard error. Differences among multiple groups were tested by unpaired two-tailed Student’s *t*-test or one-way ANOVA. Significant differences between the groups were shown as follows: * *p* < 0.05, ** *p* < 0.01, *** *p* < 0.001, and **** *p* < 0.0001.

## 5. Conclusions

In summary, a novel and promising platform for the development of oral vaccines based on *S. cerevisiae* spores was established. As a specific example, the oral vaccine of RBD displayed on the surface of spores showed perfect gastrointestinal stability, strong systematic and mucosal antibody responses, good protective capacity, and high safety. We believe that this novel platform can be used for the exploration of various oral vaccines against other potent infectious viruses or bacteria.

## Data Availability

Data is contained within the article and Supplementary Materials.

## Supplementary Materials

The following supporting information can be downloaded at: https://www.mdpi.com/article/10.3390/ijms26083615/s1.
