# Supplementary material for: Establishment of a Novel Platform for Developing Oral Vaccines Based on the Surface Display System of Yeast Spores
Source: Int J Mol Sci. 2025 Apr 11;26(8):3615. doi: 10.3390/ijms26083615 (PMC12026953; doi:10.3390/ijms26083615)
Supplement: Supplementary file 1 [file ijms-26-03615-s001.zip › ijms-3530030-supplementary.pdf]

Supplementary Materials

Establishment of a Novel Platform for Developing Oral Vaccines Based on the Surface Display System of Yeast Spores

Chenyu Si, Jiawen Bai, Yuqing Li, Yang Li, Yishi Liu, Xiaoman Zhou, Jie Shi, Hideki Nakanishi and Zijie Li *

Key Laboratory of Carbohydrate Chemistry and Biotechnology, Ministry of Education, School of Biotechnology, Jiangnan University, Wuxi 214122, China; sichenyu0402@163.com (C.S.); 18538869197@163.com (J.B.); 7210207002@stu.jiangnan.edu.cn (Y.L.); 18662776128@163.com (Y.L.); liuyishi@jiangnan.edu.cn (Y.L.); xiaoman@jiangnan.edu.cn (X.Z.); j.shi@jiangnan.edu.cn (J.S.); hideki@jiangnan.edu.cn (H.N.)

* Correspondence: lizijie@jiangnan.edu.cn

Table S1. Strains used in this study.

Strains	Characteristics
S. cerevisiae AN120 (wild-type)	*MATα/MATa ARG4/arg4-NspI his3ΔSK/his3ΔSK ho::LYS2/ho::LYS2 leu2/leu2 lys2/lys2 RME1/rme1::LEU2 trp1::hisG/ trp1::hisG ura3/ura3*
S. cerevisiae HW83 (*osw2*Δ)	*MATα/MATa ARG4/arg4-NspI his3ΔSK/his3ΔSK ho::LYS2/ho::LYS2 leu2/leu2 lys2/lys2 RME1/rme1::LEU2 trp1::hisG/ trp1::hisG ura3/ura3 osw2Δ::his5+/osw2Δ::his5+*
S. cerevisiae HW3 (*dit1*Δ)	*MATα/MATa ARG4/arg4-NspI his3ΔSK/his3ΔSK ho::LYS2/ho::LYS2 leu2/leu2 lys2/lys2 RME1/rme1::LEU2 trp1::hisG/ trp1::hisG ura3/ura3 dit1Δ::his5+/dit1Δ::his5+*
AN120/pRS306-*ss-RBD*	*S. cerevisiae* AN120 harboring pRS306-*ss-RBD*
AN120/pRS306-*ss-RBD-GFP*	*S. cerevisiae* AN120 harboring pRS306-*ss-RBD-GFP*
AN120/pRS306-*ss-RBD-HA*	*S. cerevisiae* AN120 harboring pRS306-*ss-RBD-HA*
*osw2*Δ/pRS306-*ss-RBD*	*S. cerevisiae* HW83 harboring pRS306-*ss-RBD*
*osw2*Δ/pRS306-*ss-RBD-GFP*	*S. cerevisiae* HW83 harboring pRS306-*ss-RBD-GFP*
*osw2*Δ/pRS306-*ss-RBD-HA*	*S. cerevisiae* HW83 harboring pRS306-*ss-RBD-HA*
*dit1*Δ/pRS306-*ss-CBM32-RBD*	*S. cerevisiae* HW3 harboring pRS306-*ss-CBM32-RBD*
*dit1*Δ/pRS306-*ss-CBM32-RBD-GFP*	*S. cerevisiae* HW3 harboring pRS306-*ss-CBM32-RBD-GFP*
*dit1*Δ/pRS306-*ss-CBM32-RBD-HA*	*S. cerevisiae* HW3 harboring pRS306-*ss-CBM32-RBD-HA*

Table S2. Plasmids used in this study.

Plasmids	Characteristics (5’-3’)
pRS306	Amp[r], P_{TEF1}-T_{CYCI}
pRS306-*ss*	pRS306 carrying signal peptide
pRS306-*ss-RBD*	pRS306-*ss* derivative, carrying RBD gene
pRS306-*ss-RBD-GFP*	pRS306-*ss-RBD* derivative, carrying GFP at the C terminus of RBD
pRS306-*ss-RBD-HA*	pRS306-*ss-RBD* derivative, carrying HA at the C terminus of RBD
pRS306-*ss-CBM32-RBD*	pRS306-*ss-RBD* derivative, carrying CBM32 gene at the N terminus of RBD
pRS306-*ss-CBM32-RBD-GFP*	pRS306-*ss-CBM32-RBD* derivative, carrying GFP at the C terminus of RBD
pRS306-*ss-CBM32-RBD-HA*	pRS306-*ss-CBM32-RBD* derivative, carrying HA at the C terminus of RBD

Table S3. Primers used in this study.

Primers	Characteristics (5'-3')
RBD-F	GTAAACTGTAATCCTGTTTCCAAGCTTATAAC CAATTTATGTCCATTC
RBD-R	CTCGAGGTCGACGGTATCGATAAGCTTTTAAT TCGTGGACTTTTTTGG
GFP-F	GTCCACGAATGGTGGAGGCGGTTCAGGTGGA GGTGGCTCTGGTGGTGGTGGTAGTATGGT
GFP-R	CTAATTACATGACTCGAGTTACTTGTACAGCT CGTCCATGCC
HA-F	AAAAAGTCCACGAATCTCGAGTACCCATACG ATGTTCCTGAC
HA-R	TGGCGCGCCTCAGCACTGAGCAGCGTAATCT GGAACGTCATA
CBM32-F	GTAAACTGTAATCCTGTTTCCAAGCTTAATCT TGCATTAAATAAGACC
CBM32-R	GCGCGAATTCAGATCCTCCACCACCAGAGCC ACCACCACCAGAACCACCGCCACCGCCATAG ACTTCAAATTCCCACAGT

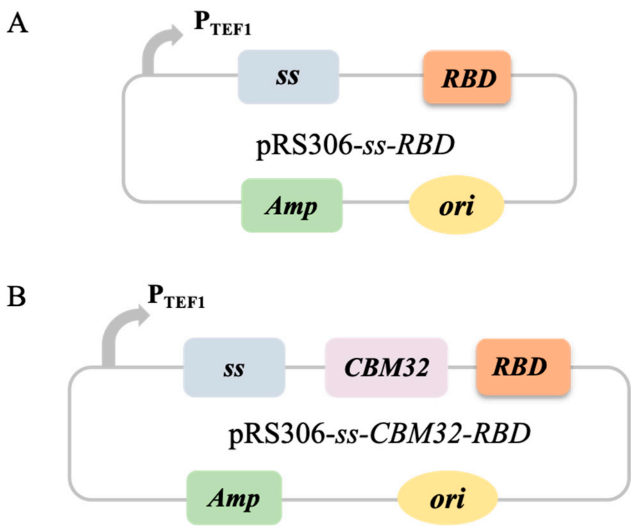

Figure S1. The maps of the recombinant constructs. (A) pRS306-*ss-RBD*. (B) pRS306-*ss-CBM32-RBD*.

The construct contains the receptor-binding domain (RBD) of SARS-CoV-2 spike protein fused to carbohydrate-binding module 32 (CBM32) under the TEF1 promoter (P_{TEF1}). Abbreviations: Amp, ampicillin resistance gene; Ori, origin of replication; ss, signal peptide sequence.

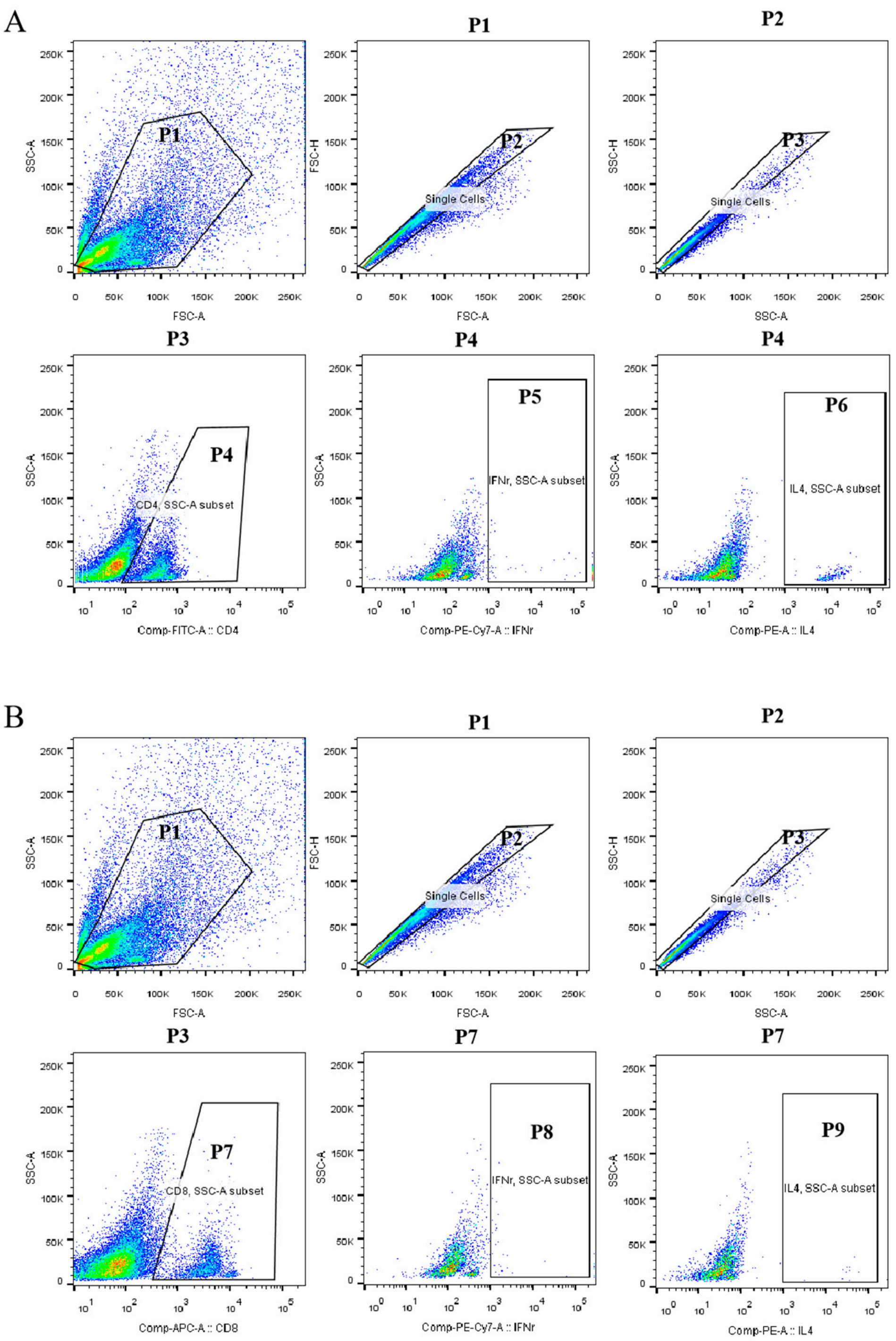

Figure S2. The FACS plot illustrating the gating strategy for identifying cytokine-producing CD8^{+} and CD4^{+} T cells. (A) CD4^{+} T cells gating strategy. (B) CD8^{+} T cells gating strategy.
